# Determination of Trace Level Iodine in Biological and Botanical Reference Materials by Isotope Dilution Mass Spectrometry

**DOI:** 10.6028/jres.094.022

**Published:** 1989

**Authors:** John W. Gramlich, Thomas J. Murphy

**Affiliations:** National Institute of Standards and Technology, Gaithersburg, MD 20899

**Keywords:** biological material, botanical material, iodine, mass spectrometry, Standard Reference Materials, trace analysis

## Abstract

A method has been developed for the determination of trace level iodine in biological and botanical materials. The method consists of spiking a sample with ^129^I, equilibration of the spike with the natural iodine, wet ashing under carefully controlled conditions, and separation of the iodine by co-precipitation with silver chloride. Measurement of the ^129^I/^127^I ratio is accomplished by negative thermal ionization mass spectrometry using LaB_6_ for ionization enhancement. The application of the method to the certification of trace iodine in two Standard Reference Materials is described.

## 1. Introduction

Iodine has been known to be necessary to human nutrition since the 19th century [1]. Endemic goiter in man was shown to be due to the lack of iodine in the food and water supply of affected regions. The addition of iodine to salt, iodized salt, has become a widely recognized factor in the control of iodine deficiency.

The Food and Nutrition Board, National Academy of Science—National Research Council has established a recommended daily allowance (RDA) of 150 *µ*g of iodine. This is the basis for the U.S. RDA published by the Food and Drug Administration for nutritional labeling of foods (Fed. Reg., March 14, 1973). Most foods contain less than 1 *µ*g/g of iodine, and considerable uncertainty exists for values of iodine determined in foods [2]. Heckman [3] published the results of an interlaboratory study of iodine in foods using neutron activation analysis and Ce-As-I colorimetry. The study showed wide discrepancies in analytical results. Mean relative standard deviations for all laboratories were almost 80%, with differences of over 300% reported between laboratories. In the worst case, reported results differed by over two orders of magnitude. The study recommended that either the chemical method be refined or a new method be developed. Dybczynski et al. [4] have reported a “round-robin” analysis of a milk powder reference material which showed iodine concentrations ranging from 0.05 to 3.6 *µ*g/g.

Until recently, none of the botanical or biological Standard Reference Materials (SRMs) issued by the National Institute of Standards and Technology were certified for iodine concentrations. A number of these SRMs contained an “information only” value provided by neutron activation analysis. However, in the absence of a second reliable analytical technique, iodine concentrations could not be certified. In view of this need for an accurate method for trace level measurements of iodine in these materials, it was decided to develop a method of analysis based on isotope dilution mass spectrometry (IDMS). Isotope dilution mass spectrometry has been used extensively at the National Institute of Standards and Technology for the certification of trace element concentrations in Standard Reference Materials. It is regarded as a “definitive method”, that is, a method for which systematic errors have been thoroughly evaluated and accounted for to provide an essentially bias free and hence accurate result.

The quantity of an element present in a material is determined by IDMS from the change produced in its isotopic composition by the addition of a known amount of a pure isotope of the element of interest. After equilibration of the element in the material with the added separated isotope of the element, followed by chemical separation, the altered isotopic composition of the element is measured by thermal ionization mass spectrometry. The concentration of the element can then be calculated from a knowledge of the sample weight and natural isotopie composition, and the weight, concentration, and isotopie composition of the added separated isotope. Although it can be used for the accurate determination of element concentrations ranging from parts-per-billion (ng/g) to percent levels, IDMS has found its greatest applicability in the certification of trace level concentrations (*µ*g/g-ng/g). It is the most accurate of the trace analysis techniques, mainly because separations need not be quantitative since only isotope ratios and not quantities are measured. For most elements, the lower limit of analysis is determined by the level of contamination or “blank.”

The development of an IDMS method for iodine presented several problems: 1) since natural iodine is a mononuclidic element (100% ^127^I), a radioactive isotope with a long half-life would have to be used as the spike isotope; 2) a method for the isotopie equilibration of the spike isotope with the natural iodine in the material, without loss of iodine before equilibration, would have to be developed; 3) a method to separate the equilibrated iodine in a form suitable for mass spectrometric analysis would be required; and 4) a mass spectrometric analytical procedure capable of providing high precision measurements on ng quantities of iodine required development.

This paper describes the development of such an IDMS method for trace iodine in botanical and biological materials, and the application of the method to the certification of iodine in botanical and biological Standard Reference Materials. This work was first reported in outline form in a 1982 report of the National Bureau of Standards [5] and is presented here in detail. Heumann and Schindlmeier [6] have published an IDMS method for the determination of iodine by negative thermal ionization mass spectrometry. These investigators used ^129^I as the spike isotope for iodine in table salt and pure inorganic chemicals. The chemical separation of the iodide ion was carried out by anion-exchange chromatography, however this method of separation could not be applied to wet ashed organic materials in a high acidity solution. Since the completion of the work described in this paper, Schindlmeier and Heumann [7] have published the determination of trace iodine in food by IDMS. Their method for decomposition of the organic matrix by wet-ashing and their mass spectrometric procedure are considerably different than those described in this paper.

In 1985 the Community Bureau of Reference (BCR) of the Commission of the Eurpoean Communities certified two milk powder samples (CRMs 150 and 151) for iodine. Various activation analysis techniques, IDMS, the catalitic Ce-As-I method, gas chromatography, and pulse voltammetry were included in the certification of these materials [8].

## 2. Experimental Section

Chemistry: Iodine-129 was selected as the spike isotope. It is a *β*-emitter with a half-life of 1.6×10^7^ years [9]. The material used was SRM 4949A, Iodine-129 Radioactivity Standard. A solution of this material was prepared in a Teflon bottle and made alkaline by the addition of sodium carbonate. The solution, calibrated against high-purity potassium iodide (E. Merck,^1^ Darmstadt, FRG), contained 0.078209±0.00011 *µ*mol/g (1 s) of iodine. The relative isotopie composition of the iodine in this spike solution was ^127^I= 13.90% and ^129^I=86.10%.

The equilibration of the spike isotope and natural ^127^I was studied using radioactive ^125^I as the tracer isotope. This isotope has a half-life of 60.1 days [9]. Sufficient spike isotope was added to each sample studied so that 3000 counts over background were observed in a 200 s time period. The *τ*-ray counting was done utilizing a 7.6×7.6 cm Nal (Tl) crystal and associated electronics. Various methods of oxidizing iodide to iodate and wet-ashing the organic matrix were investigated. It was necessary only to equilibrate the spike and natural isotopes without loss of iodine; losses after equilibration will not affect the accuracy of the analysis. Studies with ^125^I tracers and SRM 1572, Citrus Leaves, showed that fuming nitric acid solubilized the sample while retaining iodide as iodate. Wet-ashing could be effected by heating overnight on a hot-plate and then with additional heating after adding perchloric acid. Slow loss of iodine occurs at this step, but will not affect the accuracy of the results, since the spike and natural iodine are equilibrated. Co-precipitation of iodide (after reduction from iodate to iodide) was investigated using the ^125^I tracer. These experiments showed that the iodide could be co-precipitated with chloride as AgCl-AgI with 70–85% recovery of iodide.

Mass spectrometry: Several techniques and variations have been reported in the literature for negative thermal ionization of iodine. The two major approaches are multiple filament ionization using rhenium or tungsten filaments [6,7], and cataphoretic deposition of lanthanum hexaboride [10,11] to improve the sensitivity of the measurements by lowering the work function of the ionizing filament. The above references are representative of efforts in this area, and provide cross references to other published work. Each approach has advantages and disadvantages depending on the particular application. Lanthanum hexaboride coated ionizing filaments provide very high ionization efficiency and elemental sensitivity, however the deposition procedure is difficult and time consuming, and the precision of the measurements is extremely dependent upon the reproducibility of the physical characteristics of the LaB_6_ coatings. Uncoated filaments are more amenable to routine analyses but require several micrograms of iodine to obtain sufficient signal intensity.

The use of low work function filaments, using LaB_6_ to obtain the required high sensitivity for measuring ultra-trace amounts of ^129^I in the environment, has been extensively studied and reported in the literature [10,12]. Typically the ^127^I/^129^I ratio in such materials is greater than 10^6^, thus sensitivity to a small number of ^129^I atoms is of far greater importance than the precision of the measurements. With isotope dilution mass spectrometry, using a ^129^I spike, the amount of ^129^I can be adjusted to optimize the measurement of the ^127^I/^129^I ratio. Thus the reproducibility (inter and intra-analysis precision) becomes the limiting factor in the measurement of an isotopic ratio.

## 3. Procedure

Approximately 1 g of dried and accurately weighed biological or botanical material was transferred to a 250 mL Teflon-FEP screw capped bottle. The sample was spiked with the ^129^I spike solution (about 0.02 *µ*mol ^129^I for samples in the 1–3 *µ*g/g range) and 23 g (15 mL) of fuming nitric acid (90% HNO_3_, ACS Reagent Grade) were added. The bottle was capped and allowed to stand for 1 h at room temperature. The cap was then removed and a small cover glass was placed over the top of the bottle. The bottle and contents were heated at low temperature (60 °C) for approximately 1 h. The heat was then increased (75 °C) and the sample was digested overnight. The solution was allowed to cool to room temperature and 5 mL of perchloric acid (72%, ACS Reagent Grade) were added. The bottle was again covered and heated for 2 h on the hot plate at a temperature of approximately 75 °C. The cover was then removed to allow fumes of nitric oxide to escape. The solution was allowed to cool, diluted with approximately 10 mL of high purity water and transferred to a 40 mL Pyrex glass centrifuge tube. The solution was mixed and allowed to stand until it was again at room temperature. To remove any insoluble matter produced by the reactions, the tube was centrifuged at 2000 rpm for 10 min. The centrifugate was drawn off from the precipitate using a polypropylene syringe equipped with a platinium needle, and transferred to another centrifuge tube. Five mL of hydrazine sulfate solution (2 g hydrazine sulfate/100 mL, ACS reagent grade) were added and the solution was allowed to stand for 2 h. One mL of 0.01 mol/L HCl and 1 mL of 0.005 mol/L AgNO_3_ were added and the tube was allowed to stand overnight in the dark. The tube was then centrifuged at 2000 rpm for 10 min and the centrifugate was drawn off from the co-precipitated AgCl-AgI using a polypropylene syringe and platinium needle. The precipitate was washed with a few mL of high purity water, and the tube was again centrifuged at 2000 rpm for 10 min. The liquid was Withdrawn from the tube using the syringe and the platinum needle.

The precipitate was then dissolved in 200 *µ*L of an ammonium cyanide reagent solution and diluted to approximately 10 mL with high purity water. (The ammonium cyanide reagent solution was prepared by passing 50 mL of a solution containing 1 g of KCN through an acid cleaned cation exchange column and collecting the eluant containing dilute HCN in 40 mL of 2 mol/L NH_4_OH. The column was washed with 10 mL of high purity water such that the final solution contained approximately 4 mg CN^−^/mL in 1 mol/L NH_4_OH.) Ten mL of 2.5 mol/L HNO_3_ were added to re-precipitate AgCl-Agl. The tube and its contents were allowed to stand for a minimum of 2 h and then centrifuged for 10 min at 2000 rpm. The solution was drawn off the precipitate using the syringe and needle. The precipitate was washed with 0.5 mL of 0.5 mol/L NH_4_OH and allowed to stand for a few minutes. The tube was again centrifuged at 2000 rpm for 2 min and the solution was withdrawn with the syringe and needle. The precipitate was dissolved in a sufficient amount of the ammonium cyanide reagent solution to produce a solution containing approximately 10 *µ*g I/mL. This solution was transferred to a capped 1 mL centrifuge tube for mass spectrometric analysis.

The cataphoretic deposition of LaB_6_ onto rhenium filaments generally followed the procedure described by Favreau [13,14] with modifications suggested by Delmore [10,11]. The LaB_6_ (obtained from CERAC, Inc., Milwaukee, WI, lot #11610-A-1) was specified by the manufacturer for use in producing thermionic coatings on rhenium. Prior to use, the LaB_6_ (−325 mesh) was ground to a finer mesh size in an agate mortar, repeatedly washed with ethanol (ACS reagent grade), agitated in an ultrasonic cleaner, and then centrifuged. The ethanol was decanted and discarded before repeating the washing process. Although the LaB_6_ appeared to be quite clean, as received, the ethanol wash was a precaution to remove any boric oxide, as suggested by Favreau [13]. The washed LaB_6_ was dried in a vacuum oven and stored in a vacuum desiccator over magnesium Perchlorate. The solutions used for cataphoretic deposition were prepared by mixing approximately 100 mg of LaB_6_ with 10 mL of anhydrous spectroscopic grade methyl alcohol. Unlike several other reported methods, no electrolyte was added to the solutions. These solutions, if kept sealed from the atmosphere when not in use, were effective for 2 to 3 weeks.

**Figure 1:**
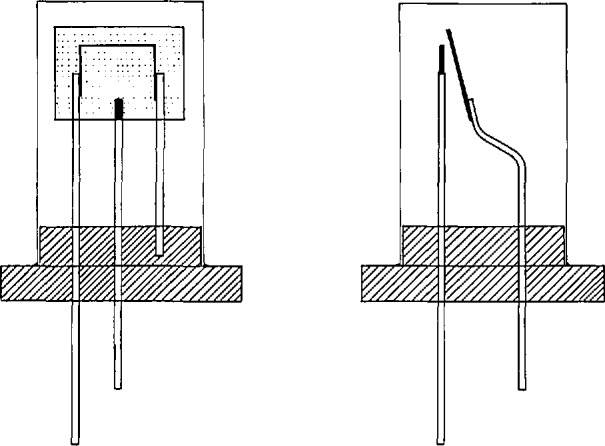
Cataphoretic deposition apparatus.

The cataphoretic deposition apparatus (fig. 1) consisted of a 15 mL borosilicate glass beaker fitted with a Teflon stopper. Gold plated connectors were installed in the bottom of the stopper to hold the ionizing filament in an inverted position in the beaker. One connector was attached to a wire extending through the stopper for electrical connection to a dc power supply. A second wire through the stopper was spot welded to a platinum foil anode (1×1×0.0076 cm). A rhenium filament, previously degassed in a vacuum and under a potential field for 1 h at a current of 4.5 A through the filament, was placed in the connectors on the bottom of the Teflon stopper. The platinum anode was aligned with the filament such that it was parallel to, and approximately 1 mm from the filament. A sufficient quantity of the LaB_6_-methyl alcohol solution was added to the beaker to submerge the filament to a depth of 1 mm. Cataphoretic deposition of LaB_6_ onto the rhenium filament was accomplished by applying a dc voltage sufficient to draw a current of 2.5 mA. This required a voltage of 60–100 V dc and was critically dependent on the spacing between the platinum anode and the filament cathode. The time of deposition ranged from 5–10 min and was based on visual judgment of the thickness of the LaB_6_ coating on the filament.

Delmore [11] has extensively studied the ionization efficiency and precision attainable with different types of LaB_6_ coatings (thick porous, thin porous and smooth dense coatings). Our work has confirmed Delmore’s investigations by showing that the porous LaB_6_ coatings provide maximum signal intensity, but the smooth dense coatings produce greater inter and intra-analysis precision at the expense of ionization efficiency. Since the materials discussed in this paper contained sufficient total iodine (sample + spike), precision rather than sensitivity was the major concern. The LaB_6_ deposition procedure described here was designed to produce a smooth dense LaB_6_ coating for optimization of the accuracy and precision of the measurements. The low work function of LaB_6_ is easily poisoned by such gases as oxygen, hydrogen, water vapor, and carbon dioxide [15,16]. Details of the poisoning mechanism have been described by Gallagher [16]. Re-activation of the LaB_6_ coated filaments was accomplished by step-wise heating in a high vacuum (1×10^−7^ Torr) at 1200, 1300, and 1400 °C for 5 min at each temperature.

Approximately 5 *µ*L (50 ng I) of the sample solution was placed on each of two previously degassed rhenium sample filaments and evaporated to dryness using a heat lamp and a current of 1 A through the filaments for 5 min. The current through the filaments was then increased until the sample deposit melted on the filament (approximately 600 °C). The two sample filaments and the LaB_6_-coated ionizing filament were then loaded into the source of the mass spectrometer. Since the LaB_6_ was re-exposed to atmospheric gases for a few minutes while being loaded into the mass spectrometer source, a short reactivation of the LaB_6_ was required. After the source had been evacuated to a pressure of 2×10^−7^ Torr the LaB_6_ was reactivated at 1400 °C for 5 min, followed by reducing the ionizing filament temperature to 1000 °C. Both sample filaments were then heated with a current of 0.25 A (time = 0 min). At 5 min into the analysis, the sample filament currents were increased to 0.50 A and at 10 min were increased to produce a total iodine ion current at the collector of 6×10^−11^ A. Ten minutes were allowed for baseline measurements and stabilization of the ion current before data collection. A stable but slightly decaying iodine signal could be maintained for several hours.

Samples for isotope dilution analysis were spiked to give approximately identical ^127^I/^129^I ratios, to minimize the effect of memory from previous samples. When analyzing samples of significantly different isotopic composition, it was necessary to clean the ion source before analysis. The effects of iodine memory in the source region must be carefully monitored to ensure accuracy.

## 4. Results and Discussion

Table 1 shows the results for the IDMS determinations of iodine in SRM 1572, Citrus Leaves. Samples were taken from six different bottles and dried for 2 h at 85 °C before the iodine was determined as described in the procedure section. The determinations were run in two sets, A and B. Two samples, 4-A and 6-A, were lost during chemical preparation and are therefore not reported. Two samples, 3-1 and 5-1, were analysed without drying and corrected to dry weight by drying separate samples. Eight “blanks” for set A averaged 45±10 ng I and three “blanks” for set B averaged 38±3 ng I. Pooling the results for the two sets produced a value of 1.835±0.008 *µ*g/g (1 s, *n* = 12) for the concentration of iodine in SRM 1572. The results of different nuclear activation analysis techniques at NIST for the determination of iodine are in good agreement with this value, though they are less precise. Analyses by instrumental photon activation analysis (IPAA), instrumental epithermal neutron activation analysis, and thermal neutron activation analysis with radiochemical separation (RNAA) found an average iodine concentration of 1.91±0.34 *µ*g/g (2 s) for this material.

Table 2 shows the results for the IDMS determination of iodine in SRM 1549, Non-Fat Milk Powder. Samples were taken from six different bottles and dried for 48 h in a vacuum at room temperature before the iodine was determined as described above. The average concentration found for iodine in SRM 1549 was 3.376±0.005 *µ*g/g (1 s, *n*=6). The blank correction for these determinations averaged 45±13 ng/g. The uncertainty in the blank correction calculates to ±0.4% of the iodine concentration in the milk powder and is probably the principal cause of uncertainty in these determinations. The results of the determination of iodine in this material by instrumental photon activation analysis and instrumental neutron activation analysis are again in good agreement with the IDMS value, however the uncertainty is much greater for the nuclear methods. Analyses by IPAA found an average iodine concentration of 3.40±0.46 *µ*g/g (2 s), while INAA obtained an average value of 3.21±0.77*µ*g/g(2 s).

## Figures and Tables

**Table 1:** Concentration of iodine in SRM 1572, Citrus Leaves

Sample No.	Bottle No.	Concentration, *µ*g/g
1-A	C-1	1.845
2-A	C-3	1.838
3-A	C-5	1.839
5-A	C-7	1.844
3-1*	C-5	1.826
5-1*	C-7	1.850
1-B	C-l	1.842
2-B	C-3	1.822
3-B	C-5	1.831
4-B	C-6	1.826
5-B	C-7	1.830
6-B	C-10	1.831
	Average = 1.835	
	s = ± 0.008	

Samples analyzed as received. Corrected to dry weight by drying separate samples.

**Table 2:** Concentration of iodine in SRM 1549, Powdered Milk

Sample No.	Bottle No.	Concentration, *µ*g/g
1	9-2	3.371
2	3-1	3.378
3	9-1	3.375
4	6-1	3.384
5	12-1	3.378
6	5-1	3.372
	Average = 3.376	
	s =±0.005	

## Footnotes

1 Certain commercial equipment, instrumentation, or materials are identified in this paper to specify adequately the experimental procedure. Such identification does not imply recommendation or endorsement by the National Institute of Standards and Technology, nor does it imply that the materials or equipment identified are necessarily the best available for the purpose.

## References

[1] Prasad AS (1978). Trace Elements and Iron in Human Metabolism.

[2] Stewart KK (1979). Nutrient Analysis of Foods: State of the Art for Routine Analysis.

[3] Heckman MM (1979). J Assoc Off Anal Chem.

[4] Dybczynski R, Veglia A, Suschny O (1980). Report on the Intercomparison Run A-11 for the Determination of Inorganic Constituents of Milk Powder.

[5] Gramlich JW, Murphy TJ (1982). Annual Report 1982.

[6] Heumann KG, Schindlmeier W, Fresenius Z (1982). Anal Chem.

[7] Schindlmeier W, Heumann KG, Fresenius Z (1985). Anal Chem.

[8] Griepink B (1986). The Additional Certification of the Content of Iodine in Two Spiked Milk Samples of Skim Milk Powder CRM 150-151.

[9] Kahn M, Kleinberg J (1977). Radiochemistry of Iodine, NAS-NS-3062.

[10] Delmore JE (1982). Int J Mass Spectrom Ion Phys.

[11] Delmore JE (1983). Rev Sci Instrum.

[12] Rankin RA, Hohorst FA, Nielsen RA, Filby EE, Emel WA (1983).

[13] Favreau LJ (1965). Rev Sci Instrum.

[14] Favreau LJ, Koenig DF (1967). Rev Sci Instrum.

[15] Buckingham JD (1965). J Appl Phys.

[16] Gallagher HE (1969). J Appl Phys.

